# Identification of Fruit-Associated QTLs in Winter Squash (*Cucurbita maxima* Duchesne) Using Recombinant Inbred Lines

**DOI:** 10.3390/genes11040419

**Published:** 2020-04-14

**Authors:** Karolina Kaźmińska, Ewelina Hallmann, Aleksandra Korzeniewska, Katarzyna Niemirowicz-Szczytt, Grzegorz Bartoszewski

**Affiliations:** 1 Department of Plant Genetics Breeding and Biotechnology, Institute of Biology, Warsaw University of Life Sciences, 02-776 Warsaw, Poland; 2 Department of Functional and Organic Food, Institute of Human Nutrition Sciences, Warsaw University of Life Sciences, 02-787 Warsaw, Poland

**Keywords:** *Cucurbita maxima*, DNA markers, fruit traits, genetic mapping, QTL

## Abstract

*Cucurbita maxima* Duchesne squash and pumpkins are cultivated world-wide. *Cucurbita maxima* fruits are produced for fresh market and are valuable for food processing. Therefore, fruit characteristics and yield are the traits of high economic importance for breeders. To date, the genetic basis of fruit-associated traits in *C. maxima* have been poorly understood. In the present study, we evaluated fruit-associated traits and conducted quantitative trait locus (QTL) analysis using recombinant inbred lines (RILs) derived from a cross of two inbred lines with different fruit morphotypes. Phenotypic data for nine fruit traits (earliness, weight, number per plant, yield per plant, length and diameter, shape index, flesh thickness, sucrose content and dry matter content) were collected for RILs in two open-field experiments. Pairwise analysis of the phenotypic data revealed correlations among the fruit and yield-associated traits. Using a previously developed genetic map, we identified 26 QTLs for eight traits. The QTLs were found in 10 locations on eight chromosomes of *C. maxima*. The QTLs were detected across experiments and explained up to 41.4% of the observed phenotypic variations. Major-effect QTLs for multiple fruit-associated traits were clustered on chromosome 4, suggesting that this genomic region has been under selection during diversification and/or domestication of *C. maxima*.

## 1. Introduction

*Cucurbita maxima* Duchesne originates from South America and has spread worldwide in the post-Columbian era. *Cucurbita maxima* is an annual species with indeterminate plant growth and a predominantly monoecious sexual system. It is an allotetraploid species with chromosome number 2*n* = 40 and a genome size estimated to be 386.8 Mb [1,2]. Currently, *C. maxima* is one of 10 Cucurbitaceae species of worldwide economic importance, and together with *C. pepo* and *C. moschata,* it is one of the three most important *Cucurbita* species [3,4,5,6]. 

*Cucurbita maxima* pumpkins, squash and gourds are commonly grown vegetables, mostly because of their fruits’ properties. *C. maxima* is recognized as the most diverse species of *Cucurbita* and its fruits are characterized by great morphological variation and differences in size, shape and peel color [7]. DNA studies have confirmed a high level of genetic diversity within *C. maxima* [8,9,10]. Fruit of the majority of *C. maxima* cultivars are consumed at full maturity and recognized as winter squashes. *Cucurbita maxima* fruit flesh is rich in carotenoids (predominantly β-carotene and lutein); sugars and starch; vitamins C and E; and fiber. Based on the fruit characteristics, seven major horticultural groups of *C. maxima* cultivars have been distinguished [11]. During domestication, *C. maxima* was selected for fruit size, and now the largest cucurbit fruits are produced by specific *C. maxima* cultivars (record fruits weigh up to 1000 kg) [6]. 

An increase in the popularity of *Cucurbita* vegetables has stimulated the breeding of new cultivars [12,13,14]. Fruit morphology, flavor and nutritional traits are targets for selection in *Cucurbita* breeding [7,15]. Thus, fruit-associated traits and fruit yield are major concerns of *C. maxima* breeders [3,12,16,17]. Modern breeding programs can be facilitated by DNA-based markers for targeted traits [7,18]. Many genes often control agronomic traits, and genomic resources, including high-density genetic maps and genome-wide association studies, can be employed to develop reliable markers for breeding. Despite biological and horticultural interest in *C. maxima*, knowledge of its genetics and genomic resources is limited in comparison with other economically important cucurbit crops. Recently, the first detailed genetic maps were developed for *C. maxima* [2,19,20,21]. Additionally, the nuclear genome of *C. maxima* cv. Rimu was sequenced and annotated [2]. Several transcriptomic studies performed on *C. maxima* focused on agrobotanical traits, which revealed gene expression patterns of sex expression, fruit development and ripening in this allotetraploid species [22,23,24]. 

Variation for traits related to fruit characteristics could be explained by a large number of quantitative trait loci (QTLs) and their interactions [25]. Esteras et al. [26] constructed a genetic map using an F_2_ population from a cross of zucchini (*C. pepo* subsp. *pepo*) with scallop (*C. pepo* subsp. *ovifera*) and identified QTLs associated with fruit-related traits. From this F_2_ population, F_8_ recombinant inbred lines (RILs) were developed, a high-density SNP-based map was constructed and 48 consistent QTLs for agronomic traits, including fruit-quality traits, were identified [27]. Zhong et al. [15] constructed a high-density SNP-based linkage map using an F_2_ population of *C. moschata* and identified 29 QTLs associated with 12 fruit traits for this species. Echevarria et al. [28] using a BC_1_ population identified several QTLs for fruit and agronomic traits in tropical pumpkin *C. moschata*. For *C. maxima*, the genetic bases of fruit-associated traits are largely unknown [4]. The only QTLs described so far for *C. maxima* have been for a dwarf vine identified in an F_2_ population [19], seed-related traits using an F_2:3_ population [21] and fruit flesh color and carotenoid content—identified using F_6_ RILs [20].

In this study, we used advanced RILs and measured several quantitative fruit-related traits of agronomic importance in open-field trials. We used a previously-developed genetic map to identify QTLs related to fruit-associated traits. This study provides us with novel data about QTLs associated with economically important traits in *C. maxima* and it contributes to the fine mapping and identification of the genes responsible for fruit traits in this cucurbit species.

## 2. Results

### 2.1. Evaluation of Earliness and Fruit-Associated Traits

Parental lines and 92 F_6_ RILs were evaluated in two open-field trials during 2013 (Exp I) and 2014 (Exp II) under Eastern European climatic conditions. The 2013 season was warmer compared with 2014 and low precipitation occurred during the flowering time of the plants (July); at the end of the vegetative season (September), the temperatures were lower and precipitation was higher as compared to 2014 and multi-year averages. The second season (2014) had more precipitation than multi-year averages during flowering time (July) while there were higher temperatures and almost no precipitation at the end of the season (September) (Supplementary Table S1). Differences in the weather conditions influenced the results of studied traits (Table 1, Supplementary Table S2). For Exp II compared to Exp I, the first female flowers appeared on average 3 days earlier in the measurement of the earliness trait, and higher values were observed for fruit weight, number, yield, length and diameter, flesh thickness and sucrose content. The content of dry matter was lower in Exp II except for parental line P_1_. The value of fruit shape index was less influenced by the growing season and was similar in both experiments (Table 1, Supplementary Figure S1). 

**Figure 1:**
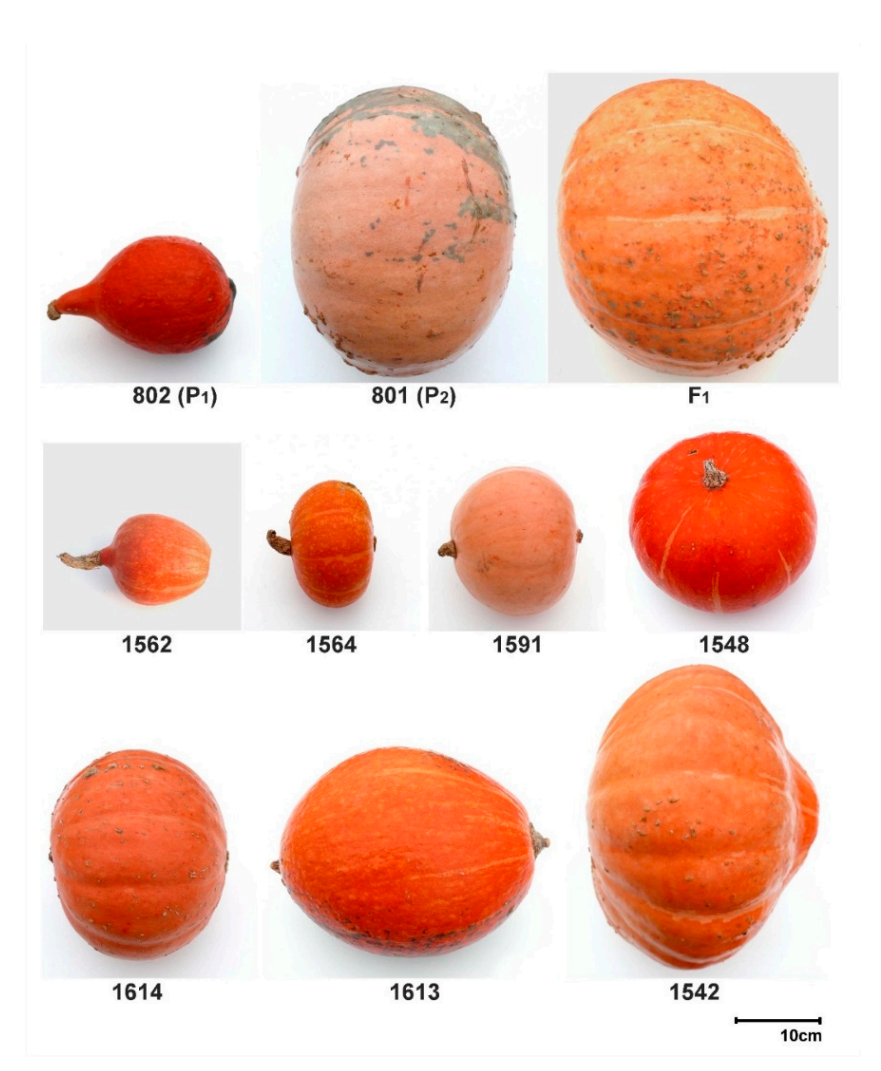
Representative fruits of the parental lines 802 (P_1_) and 801 (P_2_), and F_1_ and select F_6_ RILs (recombinant inbred lines). The pictures for experiment II are presented.

**Figure 2:**
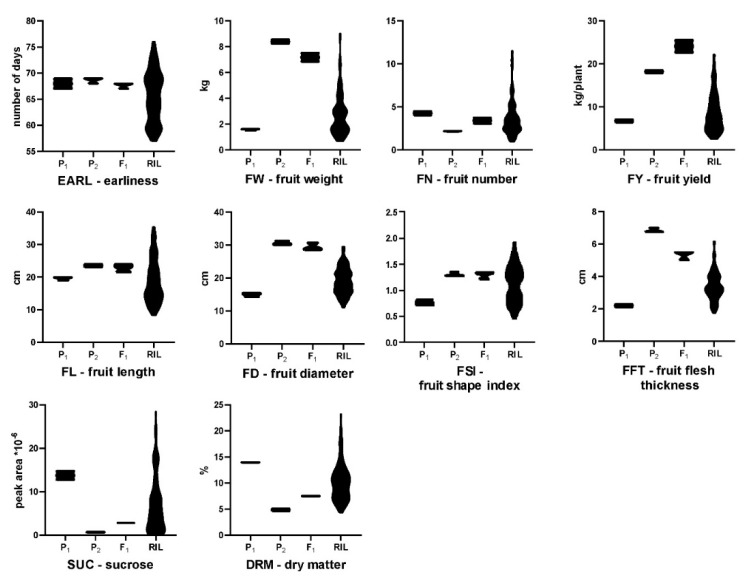
Violin plots representing the distributions of fruit-associated quantitative trait values in parental lines P_1_ and P_2_, and F_1_ and F_6_ RILs. The data for experiment II are presented.

Distribution of the examined traits among RILs departed from normality in both experiments, except for fruit diameter (Supplementary Figure S1). All the traits, except for fruit flesh thickness, showed transgressive segregation in both experiments. The values for all of the investigated traits, except for earliness, were clearly contrasting for the parental lines of the mapping population (Figure 1; Figure 2, Table 1). 

The highest broad-sense heritability values were recorded for earliness (0.95) and the lowest for fruit flesh thickness (0.46). Fruit weight and yield (0.69–0.7) and fruit number per plant (0.82) showed similar heritabilities. Heritabilities for sucrose content, dry matter content and fruit diameter were also similar (0.61, 0.57 and 0.53, respectively) (Table 1).

Pearson’s correlation coefficients were positive (>0.60) and significant for several traits. Total fruit yield per plant was positively correlated with fruit weight (0.61 and 0.63 in Exp I and Exp II, Table 2). Fruit weight was also positively correlated with fruit diameter and length and flesh thickness (values from 0.6 to 0.83). Fruit diameter was positively correlated with fruit flesh thickness (values 0.67 and 0.76). A strong positive correlation was detected between fruit length and fruit shape index (0.76 and 0.79). Moreover, a positive correlation was observed between dry matter and sucrose content (0.67 and 0.81). The strongest negative correlation was found between fruit weight and fruit dry matter (−0.5 and −0.61). Negative correlations were noted for fruit weight with fruit number and sucrose content. In addition, fruit flesh thickness was negatively correlated with sucrose and dry matter contents (from −0.34 to −0.39). In case of earliness, no significant correlations with the examined traits were detected (Table 2).

### 2.2. QTL Identification

**Figure 3:**
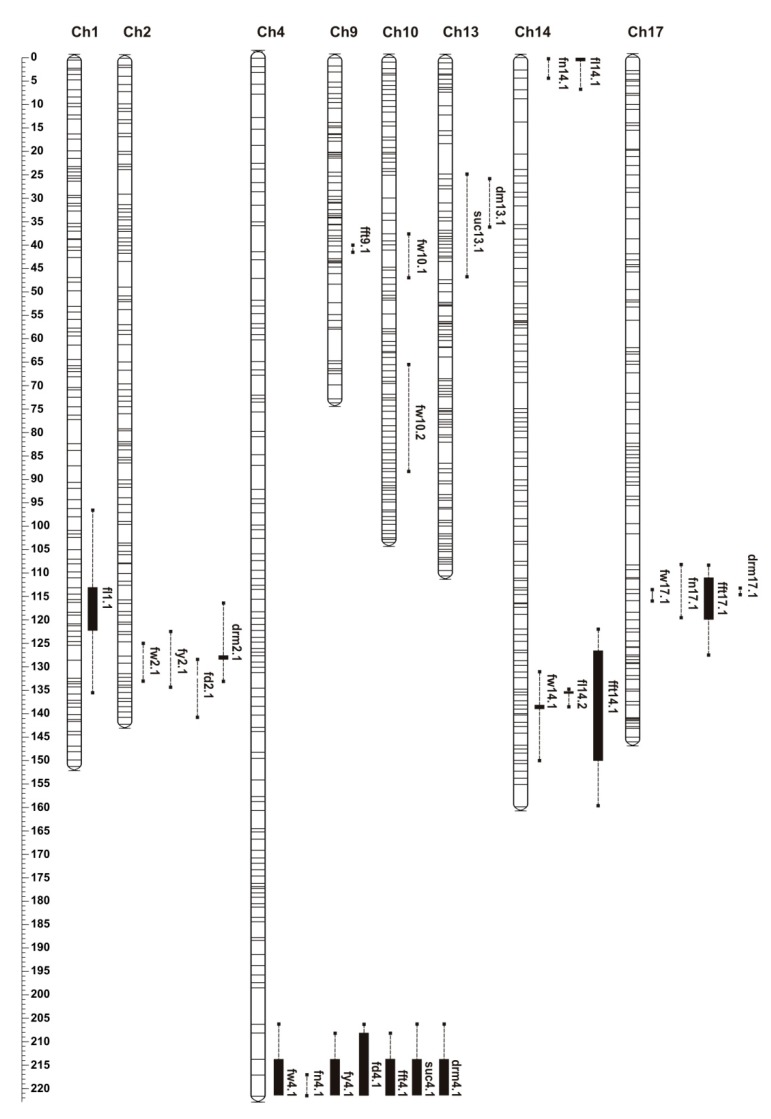
Chromosome localization of fruit-associated quantitative trait loci (QTLs) in *Cucurbita maxima*. Bars and whiskers represent identified QTLs. Black bars denote significant QTLs at *p* ≤ 0.05, while whiskers represent QTLs with maximum logarithm of odds (LOD) values below the threshold, detected in both experiments with PVE > 7%. Scale in cM.

The genetic map was constructed using 802 × 801 F_6_ RILs and consisted of 1824 markers—34 simple sequence repeat markers (SSRs), 1094 single-nucleotide polymorphism markers (SNPs) and 694 *in silico* DArTSeq markers (silicoDArTs) distributed across the 20 chromosomes of *C. maxima* [20]. A separate QTL analysis was conducted for each season’s data. In total, 26 QTLs were identified for the eight evaluated traits, including fruit weight, fruit number, yield, fruit length, fruit diameter, flesh thickness, sucrose content and dry matter content. In case of fruit shape index, no significant QTLs were detected. The parental lines did not differ significantly for earliness; hence, this trait was not included in the QTL analysis. Twenty-six QTLs were mapped to 10 different locations on *C. maxima* chromosomes (Figure 3, Table 3 and Supplementary Table S3). The properties of each QTL, including map position; logarithm of odds (LOD) threshold and LOD maximum values; percentage of phenotypic variance explained (PVE); and numbers of anchored and flanking markers are presented in Table 3. The same QTLs, but with different LOD and PVE values, were identified in both seasons for all traits. Genomic intervals corresponding to each QTL and the number of annotated genes were identified using genomic position of QTL flanking markers and *C. maxima* cv. Rimu draft genome (Supplementary Table S4).

#### 2.2.1. Fruit Weight

Parental lines differed in fruit weight, with that of the paternal line P_2_ being 4.5–5-fold higher than the one of the maternal line P_1_. The weight of F_1_ was similar to P_2_ and transgression in both directions was observed in RILs (Table 2, Figure 2). Six QTLs for fruit weight were identified. Major-effect *fw4.1* was identified on chromosome 4 (*p* ≤ 0.01) and explained 41% and 32% of the phenotypic variation in experiments I and II, respectively. QTL *fw14.1* was located on chromosome 14 (*p* ≤ 0.05) with PVE values 17.8% and 16.6% in the respective experiments. The remaining fruit weight QTLs (*fw2.1*, *fw10.1*, *fw10.2* and *fw17.1*) were detected consistently in both seasons, with lower PVE, however, ranging from 10.1% to 15.4%—and none of them exceeded the LOD threshold of *p* ≤ 0.05 (Table 3, Figure 3). 

#### 2.2.2. Fruit Number

Parental lines differed in the fruit number produced per plant—the maternal line produced on average four and the paternal line one or two fruits per season. RILs showed a positive transgressive segregation and produced from one to seven or 11 fruits per plant depending on the season (Table 1, Figure 2). Three QTLs (*fn4.1*, *fn14.1* and *fn17.1*) were identified with PVE from 9.3% to 14%; however, none of them exceeded the LOD threshold level at *p* ≤ 0.05 (Table 3). Two QTLs (*fn4.1* and *fn17.1*) were co-localized with fruit weight QTLs (Figure 3, Supplementary Table S3). As mentioned before, a negative correlation of fruit weight and fruit number was detected (Table 2). 

#### 2.2.3. Total Fruit Yield Per Plant

Differences between the parental lines in fruit weight and number were reflected in total fruit yield per plant. In F_1_, fruit yield was higher than in the parental lines, indicating the occurrence of heterosis (Table 1, Figure 2). For fruit yield per plant, two QTLs (*fy4.1* and *fy2.1*) were identified. For *fy4.1*, the LOD threshold was exceeded in Exp I, and it was slightly below the threshold in Exp II—PVEs were 24.2% and 15.8%, respectively. These two QTLs were located on chromosome 4 and 2, and they co-localized with fruit weight QTLs *fw4.1* and *fw2.1*, respectively. No fruit yield QTLs corresponding to significant fruit weight QTL *fw14.1* were found. 

#### 2.2.4. Fruit Length, Diameter and Flesh Thickness

QTL analyses for fruit length, diameter and flesh thickness revealed significant QTLs across both seasons. For fruit length, three QTLs (*fl1.1*, *fl14.1* and *fl14.2*) on chromosomes 1 and 14 were detected, and the strongest was *fl14.1* with PVE 22% in both seasons. Two loci (*fd4.1* and *fd2.1*) were identified for fruit diameter; *fd4.1* was a major-effect QTL with LOD exceeding 12.0 and PVE over 45% in both seasons. For fruit flesh thickness, four QTLs (*fft4.1*, *fft9.1*, *fft14.1* and *fft17.1*) were detected, three of which (*fft4.1*, *fft14.1* and *fft17.1*) were significant in both seasons with PVE ranging from 16.3% to 28.7% (Table 3). Interestingly, major-effect QTLs for fruit diameter *fd4.1* and fruit flesh thickness *fft4.1* were collocated at the end of chromosome 4. In this region, major-effect fruit weight and yield QTLs *fw4.1* and *fy4.1* were also identified (Figure 3, Supplementary Table S3). In addition, *fft14.1* co-localized with fruit weight QTL *fw14.1*. 

#### 2.2.5. Sucrose and Dry Matter Contents

We evaluated sucrose and dry matter contents as important fruit-quality traits. Because we evaluated mature fruits six weeks after harvest, we analyzed sucrose content only, as it is the major sugar accumulated in fruits. Growing season had a significant effect on the major sugar content, but not on the dry matter content. For the parental lines, the sucrose content was 3.9 for P_1_ and 7.8 for P_2_ times higher in Exp II than Exp I (Table 1). The range of the sucrose content in RILs showed transgressive segregation (Table 1, Figure 2). For the sucrose content, two QTLs (*suc4.1* and *suc13.1*) were identified on chromosomes 4 and 13. Major-effect *suc4.1* was significant and explained over 23% of the PVE in both seasons. For the dry matter, four QTLs (*drm2.1*, *drm4.1*, *drm14.1* and *drm17.1*) located on chromosomes 2, 4, 14 and 17 were found. QTL *drm4.1* was significant in both seasons and explained more than 27% of PVE (Table 3). Major-effect QTLs for the sucrose and dry matter contents *suc4.1* and *drm4.1* were co-localized on chromosome 4 with major-effect QTLs for fruit weight *fw4.1*, yield *fy4.1*, diameter *fd4.1* and flesh thickness *fft4.1* (Figure 3, Supplementary Table S3). 

## 3. Discussion

This study describes the identification of QTLs for important fruit traits of *C. maxima,* which is the most diverse species of *Cucurbita,* characterized by fruit morphological complexity [7,11]. Certain cultivars of *C. maxima* produce one of the biggest fruits among all plants, which makes this cucurbit an interesting model plant to investigate genetic mechanisms underlying fruit size and its evolution [6,23]. In this study, advanced F_6_ RILs were used to investigate nine agronomically important fruit-associated traits (Figure 1, Table 1). For most of the traits, transgressive segregation in both directions was observed, suggesting that crossing among *C. maxima* from different horticultural groups could be used to explore transgression for earliness and fruit-related traits to develop novel germplasm. In our study, several positive and negative correlations of fruit-associated traits were found. Correlations related to fruit weight and yield and fruit number per plant were revealed. Additionally, a negative correlation between fruit weight and sucrose and dry matter contents was noted. That suggests that selection for small-sized fruit would favor high sugar and dry matter content in the fruit flesh. Recently, consumers prefer winter squash varieties characterized by small size fruit (personal size) with firm, sweet and orange flesh. In our study, fruit length was positively correlated with fruit shape index confirming that fruit elongation plays an important role in the determination of *C. maxima* fruit shape. In cucumber, it was demonstrated that fruit length is highly correlated with fruit shape index [29]. Despite different fruit morphology in *C. maxima*, it seems that fruit length is also a key component of fruit shape index in this cucurbit crop. However, in our investigation, fruit shape index was calculated as FL divided by FD ratio, and we think that this formula did not reflect, accurately, the great variation of fruit shape appearing in studied RILs (Figure 1; Figure 2). This could be the reason why we did not identify significant QTL(s) for fruit shape. We believe that application of more sophisticated approach for fruit shape phenotyping will help to map fruit shape QTL(s) in *C. maxima*.

For all of the studied traits, except earliness, considerable differences between parental lines were expected and indeed observed. The highest-effect QTL identified in this study was *fw4.1* for fruit weight mapping to the end of chromosome 4. In this region, major-effect QTLs were also mapped for fruit yield, diameter, flesh thickness and sucrose and dry matter contents. In the same region, major-effect QTLs for fruit color and carotenoid content were identified [20]. Co-localization of major-effect QTLs for fruit characteristics in a single region at the end of chromosome 4 could be explained by *C. maxima* diversification and/or domestication processes. As mentioned by Ferriol and Picó [7], fruit size and color were under selection during those processes. In many cases, domestication-related traits are controlled by a few relatively strong QTLs that tend to be clustered [30]. Similarly, in *C. moschata* major-effect QTLs for pericarp color, carotenoids content and lutein and α-carotene contents shared the same region of linkage group LG8 with strong QTLs for fruit morphological traits—fruit diameter, pulp thickness and chamber width [15].

Sugars are an important fruit-quality trait of *C. maxima* [31], and during fruit development starch is predominantly accumulated in the fruit; however, sucrose is a major sugar present in post-harvest-stored fruits [32,33]. In our study, we analyzed sucrose content in fruit stored for 6 weeks, and considerable differences between both parents in sucrose content were observed (Table 1). We identified two QTLs (*suc4.1* and *suc13.1*) associated with sucrose content in fruit. The major one, *suc4.1*, with PVE 23.4% and 23.9% in Exp I and II, respectively, was reproducible in both years. The second one, *suc13.1*, with PVE 14.5% and 7.1%, was not stable over the two years. In *C. moschata*, Zhong et al. [15] identified a single QTL for sucrose content with PVE 11.3%; however, the fruits were harvested 45 days after anthesis, and they were not stored as long as in this study. Seroczyńska et al. [31,34] investigated dry matter contents in several lines and cultivars of *C. maxima,* which ranged from 3% to 25.1%. The results of this study are within this range, with over 2.3 times higher dry matter content in the parental line P_1_ 802. This study detected QTLs for dry matter content, major-effect *drm4.1*, with PVE 27.1% and 29.9% in Exp 1 and 2, respectively, and three with less stable effects (*drm2.1, drm13.1* and *drm17.1*) with lower PVE values (Table 3). 

Several genes underlying the QTLs related to fruit size and shape have been identified in tomatoes and cucurbits [25,29,35]. In our study, the genomic regions carrying mapped QTLs had a large number of genes, making it difficult to identify specific candidates responsible for fruit-associated traits in *C. maxima* (Supplementary Table S4). Additionally, genome sequences of the parental lines are not available. In our study, major-effect QTLs for fruit-associated traits were co-localized at the end of chromosome 4 (*fw4.1*, *fd4.1*, *fy4.1*, *fft4.1*, *fn4.1*, *suc4.1* and *drm4.1*) (Table 3, Figure 3). Within the *C. maxima* genomic region corresponding to this interval, we found gene encoding of an IQ-domain containing protein (IQD) (CmaCh04G026000.1). This gene could be possibly proposed as a candidate gene underlying major fruit-associated QTLs found at the end of chromosome 4. In tomato, the *SUN* gene encoding an IQD protein is responsible for elongated fruit shape [36,37]. Similarly, in cucumbers and watermelons, *SUN* homologs were proposed as candidate genes underlying fruit-shape QTLs [38,39]. We found *SUN* homologs (CmaCh01G017290.1 and CmaCh14G017420.1) in the genomic regions corresponding to significant QTLs for fruit length and flesh thickness (*fl1.1* and *fft14.1*). Interestingly, we also found a putative *OVATE FAMILY PROTEIN* (*OFP*) gene (CmaCh14G017080.1) in the genomic region corresponding to fruit weight and flesh thickness QTLs identified on chromosome 14 (*fw14.1* and *fft14.1*). In *C. pepo*, the putative *OFP* gene was found in the genomic region for major-effect fruit shape QTLs on LG3 [26]. In the same region in *C. pepo*, a gene encoding YABBY-like transcription factor was found too. In our study, in contrast to *C. pepo*, *YABBY* family member (CmaCh02G015570.1) was found on chromosome 2, on which minor QTLs for fruit diameter, weight, yield and dry matter content (*fd2.1*, *fw2.1*, *fy2.1* and *drm2.1*) were located. Further work and fine-mapping are needed to identify and validate gene(s) responsible for identified QTLs. Primary candidates for fine mapping and cloning of the gene(s) will be the significant major-effect QTLs located at the end of chromosome 4.

## 4. Materials and Methods 

### 4.1. Plant Material

Mapping population was developed by crossing two highly-inbred (>S_12_), monoecious, non-determinate lines of *C. maxima*, representing two contrasting fruit morphotypes (Figure 1). Line 802 (P_1_, maternal line) was derived from cultivar “Uschiki Kuri” (Hubbard group), characterized by small, pear-shaped fruit with hard, orange flesh. Line 801 (P_2_, paternal line) was derived from an Eastern European landrace originating from the former Soviet Union with large, slightly flattened fruit and soft, pale orange flesh (not classified to a major horticultural group). Parental lines of RILs were genetically distant [10] and differed in fruit-associated traits (Table 1, Figure 1; Figure 2). Ninety-two RILs were developed by a single-seed descent to the F_6_ generation. RILs showed the variability of fruit phenotypes (Figure 1; Figure 2). This RILs population has been used in a previous study for QTL mapping of fruit flesh color and carotenoid content [20].

### 4.2. Field Trials 

Observations and evaluations of the parental lines and F_6_ RILs were assessed in two open-field experiments at the Wolica Experimental Station of the Department of Plant Genetics, Breeding and Biotechnology, Warsaw, Poland. The experiments were performed in growing seasons 2013 and 2014 (Exp I and II, respectively). The seasons differed between each other in temperature and precipitation (Supplementary Table S1). The experiments were arranged in a randomized complete block design with three replicates for each line; hence, there were three plots for each line with six plants per plot. Seeds were sown directly into the soil on May 15 at a spacing of 1.2 × 1.6 m. No irrigation or pesticides were used. Fruits, harvested at the beginning of October at the stage of 70–80 days after first flower anthesis (70–80 DAA), were stored for 6 weeks until mid-November in a plastic tunnel at temperatures of 5–10 °C and then used for phenotyping. 

### 4.3. Fruit Phenotyping, Sucrose Content and Dry Matter Content Evaluation

Nine traits were evaluated in both open-field experiments. Earliness (EARL) was evaluated as a number of days from seed sowing to appearance of the first female flower. Measured fruit-associated traits included: fruit weight (FW), fruit number per plant (FN), total fruit yield per plant (FY), fruit diameter (FD), fruit length (FL) and fruit flesh thickness (FFT). Fruit shape index (FSI) as a FL-to-FD ratio was calculated. For sucrose (SUC) and dry matter (DRM) content measurements, fruit tissue was sampled as described before [20]. Briefly, for each line, six uniform fruits from each of replication were chosen and a sample was taken from the sunny site of each fruit. After removing the skin and seeds, fruit flesh samples were taken and pooled—one pooled sample per replication. All samples were stored at –80 °C until use. Samples (5 g) were homogenized in liquid nitrogen (1:20 w/v ratio) and 100 mg of the homogenate was used for sugar extraction. Sugar extraction and HPLC measurements were performed as described by Ponder and Hallmann [39]. Sucrose content was estimated based on clearly distinguishable peaks as the peak area per microgram of fresh weight. Dry matter (percentage of fresh matter) was determined by drying 5 g samples at 105 °C to a constant mass. All phenotypic evaluation data are included in Supplementary Table S2.

### 4.4. QTL Identification

QTLs were placed on the *C. maxima* genetic map developed in a previous study, which consisted of 1824 molecular markers (34 SSRs, 1094 SNPs and 694 silicoDArTs) distributed across 20 chromosomes of *C. maxima* [20] (Supplementary Table S3). QTLs were detected using MapQTL 5.0 software and interval mapping (IM) [40]. logarithm of odds (LOD) thresholds were determined by permutation analysis based on 1000 permutations at *p* ≤ 0.05 and *p* ≤ 0.01 significance levels. QTLs exceeding the threshold value (*p* ≤ 0.05) were considered as significant. The percentage of the phenotypic variance explained by QTLs (PVE, *R*^2^) was estimated at the highest probability peak. The draft of the *C. maxima* genome sequence [2] available at Cucurbit Genomics Database (http://cucurbitgenomics.org/) [41] was used to identify genomic regions corresponding to each QTL. Sequences of the markers flanking QTLs were aligned by using basic local alignment search tool (BLAST) to the *C. maxima* cv. Rimu v1.1 genome sequence, and genomic regions corresponding to QTL intervals were revealed (Supplementary Table S4). In order to search genomic intervals for genes responsible for fruit size and shape determination, sequences of the genes responsible for fruit-associated QTLs, described by Monforte et al. [25] were used. Sequences collected by Monforte et al. ([25], supplementary Table S2) were aligned by using BLAST to the sequences of identified genomic intervals of *C. maxima*. Next, annotation of the *C. maxima* reference genome was checked to validate BLAST results to reveal putative candidate genes possibly responsible for identified QTLs. 

### 4.5. Statistical Analysis 

For each of the studied traits, histogram were prepared. Violin graphs were drawn using GraphPad Prism version 8.0 (GraphPad Software, San Diego, CA, USA). Normal distribution of each trait in the mapping population was tested by the Shapiro–Wilk test. Correlations between traits were calculated using Pearson’s correlation coefficient. All statistical analyses were performed using Statistica 12 software (StatSoft Inc., Tulsa, OK, USA). Broad sense heritability was estimated using the variance component method [42] with the following formula H^2^ = σ^2^g/σ^2^p, where σ^2^g = genotypic variance and σ^2^p = phenotypic variance [43].

## 5. Conclusions

In summary, we investigated and identified significant QTLs associated with *C. maxima* fruit traits, paving the way for fine-mapping and cloning of candidate genes affecting fruits. To our knowledge, it is the first attempt to determinate genetic bases of fruit-associated traits in this species, which is known to produce extreme-sized fruits. 

## Figures and Tables

**Table 1:** Summary of the trait evaluation for parental lines P_1_ 802 and P_2_ 801, and F_1_ and 92 F_6_ recombinant inbred lines (RILs) in 2013 and 2014 (experiments I and II). Mean values, standard deviation, minimum and maximum values and broad sense heritability value (H^2^) are presented.

Trait (Unit)	Experiment	Parental Lines		F_1_	RILs		H^2^
		P_1_ 802	P_2_ 801		Mean	Range	
Earliness (days) EARL	I	74 ± 0.6	71 ± 0.0	-	68	53–80	-
	II	68 ± 1.0	69 ± 0.6	67 ± 0.6	65	56–76	0.95
Fruit weight (kg) FW	I	1.1 ± 0.0	4.9 ± 0.0	-	2.5	0.6–5.7	-
	II	1.6 ± 0.1	8.4 ± 0.4	7.2 ± 0.4	2.9	0.7–9.0	0.7
Fruit number FN	I	4.0 ± 0.0	1.6 ± 0.3	-	2.3	1.0–7.5	-
	II	4.3 ± 0.8	2.2 ± 0.0	3.4 ± 0.9	3.6	1.0–11.5	0.82
Fruit yield (kg/plant) FY	I	4.3 ± 0.1	7.7 ± 1.2	-	4.9	1.7–13.0	-
	II	6.7 ± 1.5	18.2 ± 0.8	24.1 ± 2.1	8.8	2.7–24.1	0.69
Fruit length (cm) FL	I	16.3 ± 0.8	20.4 ± 0.6	-	18.2	8.0–36.3	-
	II	19.6 ± 0.5	23.5 ± 0.5	22.8 ± 1.3	18.8	8.4–35.3	0.92
Fruit diameter (cm) FD	I	13.1 ± 0.6	25.6 ± 1.1	-	19.2	10.0–28.1	-
	II	14.9 ± 0.6	30.6 ± 0.7	29.4 ± 1.2	19.3	11.0–30.6	0.53
Fruit shape index (FL/FD) FSI	I	1.3 ± 0.0	0.8 ± 0.0	-	1.0	0.4–2.0	-
	II	1.3 ± 0.1	0.8 ± 0.1	0.8 ± 0.1	1.0	0.5–2.2	0.71
Fruit flesh thickness (cm) FFT	I	1.8 ± 0.0	5.9 ± 0.1	-	3.2	1.8–5.9	-
	II	2.0 ± 0.0	6.8 ± 0.1	5.3 ± 0.3	3.5	1.8–6.8	0.46
Sucrose content (peak area*10^−6^) SUC	I	3.5 ± 0.5	0.9 ± 0.3	-	7.2	0.6–26.0	-
	II	13.7 ± 1.5	6.7 ± 1.7	2.8 ± 0.1	7.3	0.2–28.4	0.61
Dry matter content (%) DRM	I	12.7 ± 0.2	5.5 ± 0.2	-	11.4	4.3–19.9	-
	II	14.0 ± 0.0	4.9 ± 0.3	7.5 ± 0.1	10.3	4.3–20.2	0.57

**Table 2:** Pearson’s correlation coefficient analysis of fruit-related traits in experiments 1 and 2 (Exp I and II). EARL—earliness; FW—fruit weight; FN—fruit number; FY—fruit yield; FL—fruit length; FD—fruit diameter; FSI—fruit shape index; FFT—fruit flesh thickness; SUC—sucrose content; DRM—dry matter content.

Experiment I										
	EARL	FW	FN	FY	FL	FD	FSI	FFT	SUC	DRM
EARL	1									
FW	0.02	1								
FN	0.01	−0.60 *	1							
FY	0.09	0.63 *	0.07	1						
FL	−0.10	0.63 *	−0.39 *	0.38 *	1					
FD	0.07	0.83 *	−0.54 *	0.58 *	0.31 *	1				
FSI	−0.13	0.11	−0.03	0.04	0.79 *	−0.30 *	1			
FFT	0.04	0.60 *	−0.45 *	0.27 *	0.20 *	0.67 *	−0.23 *	1		
SUC	−0.10	−0.50 *	0.22 *	−0.38 *	−0.32 *	−0.51 *	−0.02	−0.38 *	1	
DM	−0.10	−0.61 *	0.21 *	−0.54 *	−0.41 *	−0.56 *	−0.06	−0.39 *	0.67 *	1
Experiment II										
	EARL	FW	FN	FY	FL	FD	FSI	FFT	SUC	DRM
EARL	1									
FW	−0.05	1								
FN	0.10	−0.48 *	1							
FY	0.04	0.61 *	0.21 *	1						
FL	0.00	0.67 *	−0.41 *	0.36 *	1					
FD	−0.05	0.82 *	−0.40 *	0.65 *	0.33 *	1				
FSI	0.04	0.11	−0.14	−0.06	0.76 *	−0.33 *	1			
FFT	0.04	0.81 *	−0.46 *	0.51 *	0.52 *	0.76 *	0.00	1		
SUC	0.11	−0.45 *	0.14	−0.42 *	−0.25 *	−0.51 *	0.11	−0.34 *	1	
DM	0.13	−0.50 *	0.23 *	−0.39 *	−0.23 *	−0.52 *	0.14	−0.39 *	0.81 *	1

* significant at *p* < 0.05.

**Table 3:** The list of quantitative trait loci (QTLs) identified for fruit-associated traits in *Cucurbita maxima* 802 × 801 F_6_ RILs. PVE: percentage of phenotypic variance explained. Maximum logarithm of odds (LOD) values for significant QTLs are marked with star. Other QTLs were stable in both trials and explained more than 7% of phenotypic variance, but the maximum LOD score was below the threshold.

Trait	QTL	Chromosome	Trial	Map Position (cM)	LOD Threshold		LOD Max	PVE Max	No. of Anchored Markers	Flanking Markers	
					*p* ≤ 0.05	*p* ≤ 0.01					
Fruit weight	*fw2.1*	2	I	124.6−133.0	3.8	4.7	3.42	15.4	6	2−4931959	2−is20583086
			II	127.6	3.5	4.1	2.18	10.1	1	5139555	−
	*fw4.1*	4	I	207.5−222.9	3.8	4.7	10.92 *	41.4 *	5	4−is20582998	4−4930790
			II	207.5−222.9	3.5	4.1	7.88 *	32.0 *	5	4−is20582998	4−4930790
	*fw10.1*	10	I	38.3−47.9	3.8	4.7	2.63	12.1	7	10−4931323	10−is20583259
			II	39.9−47.9	3.5	4.1	2.51	11.9	6	10−is4928649	10−is20583259
	*fw10.2*	10	I	66.8−90.1	3.8	4.7	2.59	12.9	21	10−is20585534	20584424
			II	66.8−90.1	3.5	4.1	2.78	12.7	21	10−is20585534	20584424
	*fw14.1*	14	I	133.7−152.9	3.8	4.7	3.89 *	17.8 *	17	14−5139799	14−4929459
			II	133.7−152.9	3.5	4.1	3.55 *	16.6 *	17	14−5139799	14−4929459
	*fw17.1*	17	I	115.9−118.2	3.8	4.7	3.25	14.7	3	17−4931046	is20586067
			II	115.9−118.2	3.5	4.1	3.17	14.4	3	17−4931046	is20586067
Fruit number	*fn4.1*	4	I	218.2−222.9	3.4	3.9	2.39	11.1	2	4−20584618	4−4930790
			II	218.2−222.9	3.3	3.8	2.25	10.4	2	4−20584618	4−4930790
	*fn14.1*	14	I	0.0−4.5	3.4	3.9	1.99	9.3	3	14−20584155	14−is20583171
			II	0.0	3.3	3.8	2.33	10.8	1	14−20584155	−
	*fn17.1*	17	I	110.5−122.5	3.4	3.9	3.09	14.0	9	17−is20583488	17−20586027
			II	111.5−116.7	3.3	3.8	2.81	12.9	5	17−is20584507	17−4929849
Fruit yield	*fy2.1*	2	I	122.3−134.3	3.5	4.3	3.19	14.5	10	2−20585893	2−4931998
			II	127.6	3.7	4.2	2.16	10.0	1	5139555	−
	*fy4.1*	4	I	209.3−222.9	3.5	4.3	5.66 *	24.2 *	4	4−20584045	4−4930790
			II	209.3−222.9	3.7	4.2	3.51	15.8	4	4−20584045	4−4930790
Fruit length	*fl1.1*	1	I	97.9−135.6	3.7	4.6	3.0	13.7	27	1−4927658	1−20585139
			II	96.2−132.4	3.8	4.6	3.99 *	17.7 *	24	1−is20585554	1−20584470
	*fl14.1*	14	I	0.0−7.0	3.7	4.6	5.21 *	22.5 *	4	14−20584155	14−4929364
			II	0.0−7.0	3.8	4.6	5.18 *	22.4 *	4	14−20584155	14−4929364
	*fl14.2*	14	I	137.3−141.7	3.7	4.6	3.51	15.8	6	14−4932159	14−20585540
			II	137.3−141.7	3.8	4.6	3.9 *	17.4 *	6	14−4932159	14−20585540
Fruit diameter	*fd2.1*	2	I	127.6−140.6	3.7	4.5	3.48	15.7	13	5139555	2−is20585890
			II	127.6−133.0	3.6	4.6	2.72	12.5	5	5139555	2−is20583086
	*fd4.1*	4	I	207.5−222.9	3.7	4.5	13.15 *	47.5 *	5	4−is20582998	4−4930790
			II	207.5−222.9	3.6	4.6	12.37 *	45.4 *	5	4−is20582998	4−4930790
Fruit flesh thickness	*fft4.1*	4	I	218.3−222.9	3.6	4.1	3.63 *	16.3 *	2	4−20584618	4−4930790
			II	209.3−222.9	3.6	4.3	6.89 *	28.7 *	4	4−20584045	4−4930790
	*fft9.1*	9	I	52.2	3.6	4.1	2.45	11.5	1	9−is4927472	−
			II	52.2−52.6	3.6	4.3	2.39	11.3	2	9−is4927472	9−is4927534
	*fft14.1*	14	I	124.3−163.1	3.6	4.1	4.23 *	19.5 *	29	14−4928785	*ovc*
			II	128.9−152.9	3.6	4.3	3.8 *	17.7 *	21	14−4928237	14−4929459
	*fft17.1*	17	I	115.9−122.5	3.6	4.1	3.75 *	16.8 *	5	17−4931046	17−20586027
			II	110.5−128.5	3.6	4.3	5.24 *	22.6 *	15	17−is20583488	17−4928426
Sucrose content	*suc4.1*	4	I	207.5−222.9	3.5	4.1	5.44 *	23.4 *	5	4−is20582998	4−4930790
			II	207.5−222.9	3.7	4.5	5.58 *	23.9 *	5	4−is20582998	4−4930790
	*suc13.1*	13	I	14.9−36.5	3.5	4.1	3.15	14.5	20	20585970	13−is20583168
			II	16.3	3.7	4.5	1.5	7.1	1	20586134	−
Dry matter content	*drm2.1*	2	I	118.1−133.0	3.7	4.4	3.82 *	17.1 *	12	2−is20583484	2−is20583086
			II	118.1−120.8	3.6	4.2	2.71	12.5	4	2−is20583484	20585633
	*drm4.1*	4	I	207.5−222.9	3.7	4.4	7.26 *	29.9 *	5	4−is20582998	4−4930790
			II	207.5−222.9	3.6	4.2	6.46 *	27.1 *	5	4−is20582998	4−4930790
	*drm13.1*	13	I	16.3−24.5	3.7	4.4	1.59	7.5	8	20586134	is20583254
			II	16.3−24.5	3.6	4.2	2.98	13.6	8	20586134	is20583254
	*drm17.1*	17	I	115.9−116.7	3.7	4.4	2.32	10.7	2	17−4931046	17−4929849
			II	115.9−116.7	3.6	4.2	1.83	8.6	2	17−4931046	17−4929849

* significant at *p* ≤ 0.05.

## Supplementary Materials

Supplementary materials can be found at https://www.mdpi.com/2073-4425/11/4/419/s1. Figure S1. Frequency distribution of the values for quantitative traits in two-year experiments (Exp I and Exp II). A—earliness (EARL), B—fruit weight (FW), C—fruit number (FN), D—fruit yield (FY), E—fruit length (FL), F—fruit diameter (FD), G—fruit shape index (FSI), H—fruit flesh thickness (FFT), sucrose content (SUC) and dry matter (DRM) content. Figure S2: HPLC profiles of measured sugar for parental lines and F_1_ in Exp 2. Peak represents: (1) fructose, (2) glucose, (3) sucrose. A—P_1_-802, B—P_2_-801 and C—F_1_. Table S1: Mean air temperature and precipitation sums in the vegetation period of *C. maxima*. Table S2: Phenotypic data of agronomic traits. Table S3: Genetic map of *C. maxima* developed by Kaźmińska et al. (2018) with fruit-associated QTLs positions (n.d.—not determined, marker not found in the genome). Table S4: Genomic regions corresponding to QTLs intervals (QTLs intervals identified in two seasons).
