# Supplementary material for: Identification of Fruit-Associated QTLs in Winter Squash (Cucurbita maxima Duchesne) Using Recombinant Inbred Lines
Source: Genes (Basel). 2020 Apr 14;11(4):419. doi: 10.3390/genes11040419 (PMC7230694; doi:10.3390/genes11040419)

# Supplementary Figures S1-S2

# Manuscript title: Identification of fruit-associated QTLs in winter squash (*Cucurbita maxima* Duchesne) using recombinant inbred lines

Authors: Karolina Kaźmińska^A^, Ewelina Hallmann^B^, Aleksandra Korzeniewska^A^, Katarzyna Niemirowicz-Szczytt^A^, Grzegorz Bartoszewski^A^*

^A^Department of Plant Genetics Breeding and Biotechnology, Warsaw University of Life Sciences, Warsaw, Poland

^B^ Department of Functional and Organic Food, Institute of Human Nutrition Sciences, Warsaw University of Life Sciences, Warsaw, Poland

*Correspondence to grzegorz_bartoszewski@sggw.pl

# Supplementary Figure S1.

Frequency distribution of the values for quantitative trait in two-year experiments (Exp I and Exp II). A – earliness (EARL), B – fruit weight (FW), C – fruit number (FN), D – fruit yield (FY), E – fruit length (FL), F – fruit diameter (FD), G – fruit shape index (FSI), H – fruit flesh thickness (FFT), sucrose (SUC) and dry matter (DRM) content.


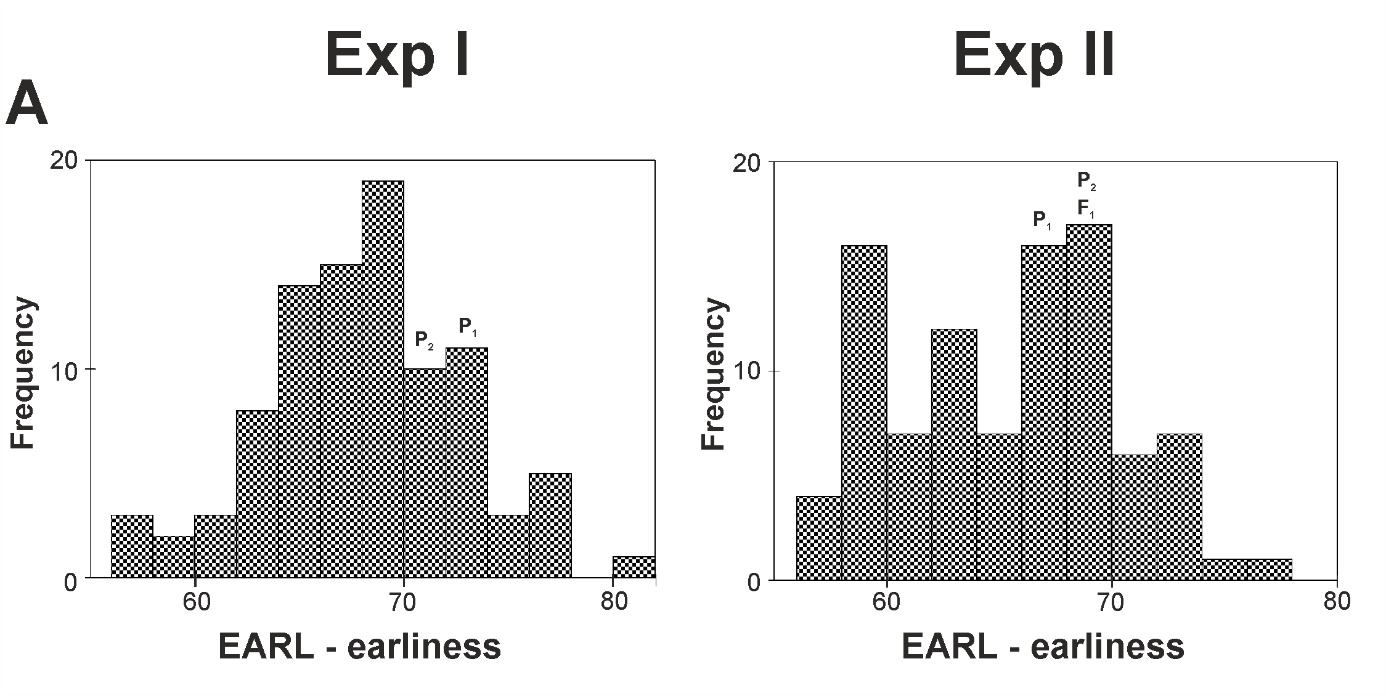


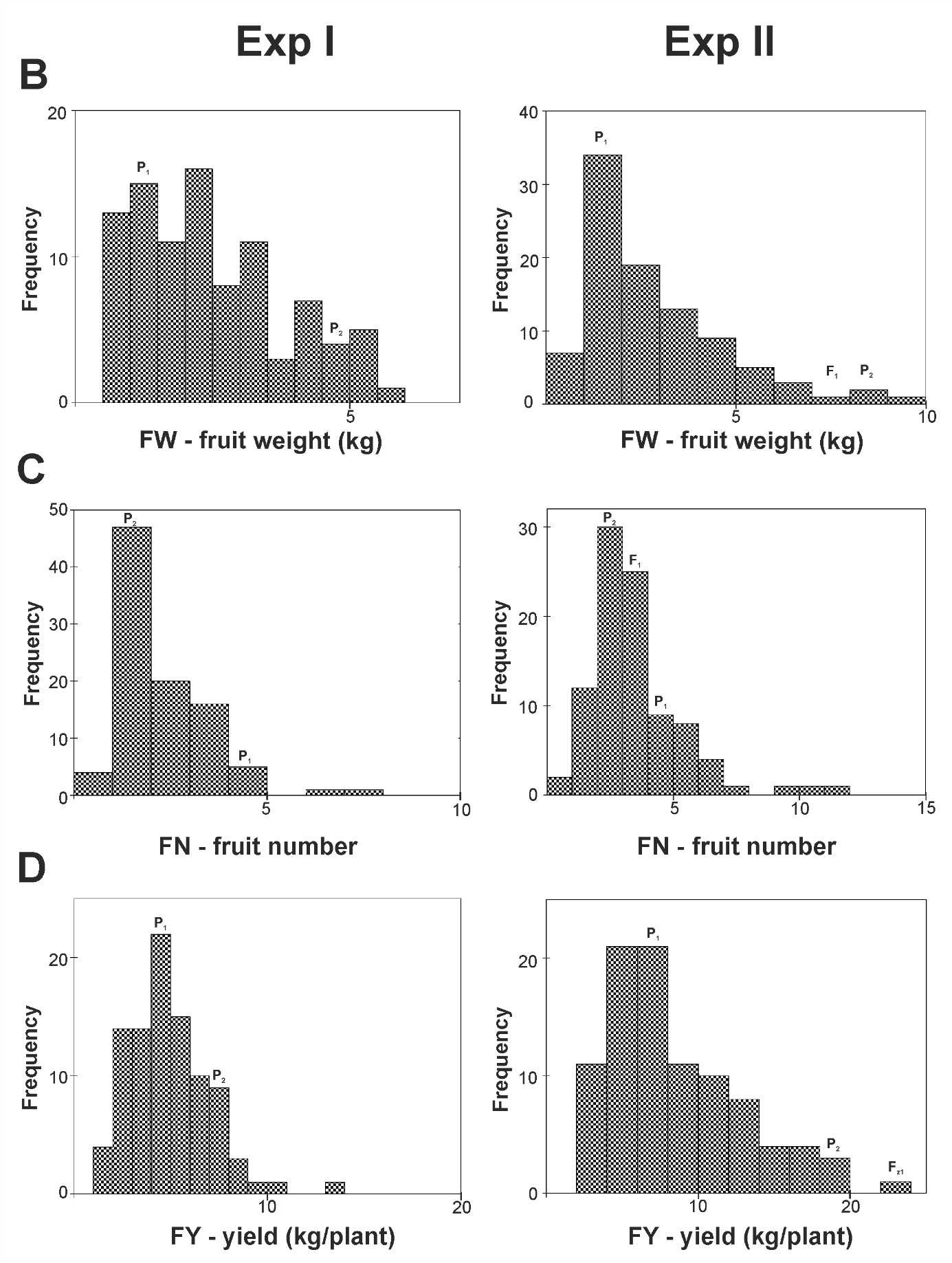

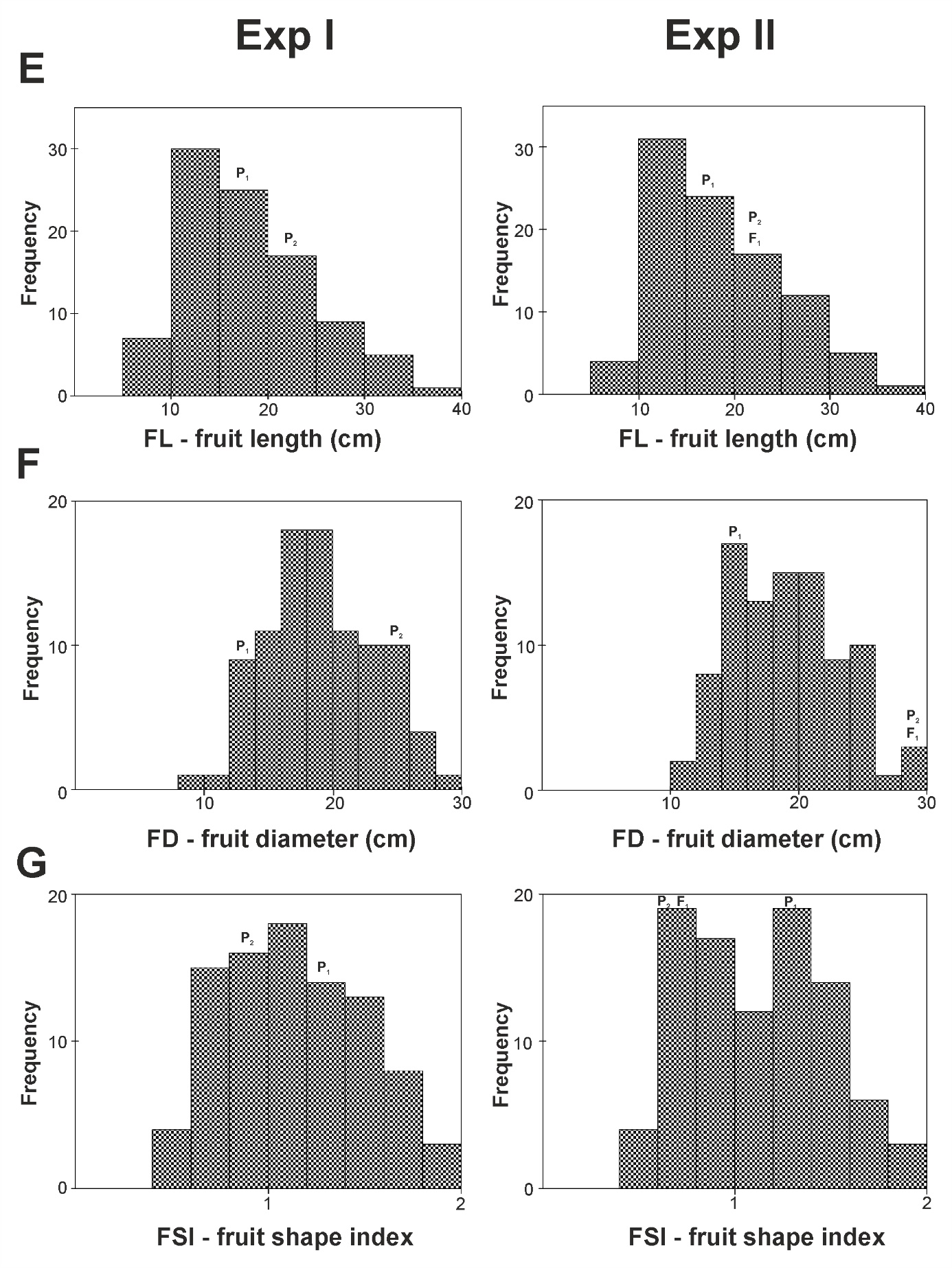
x


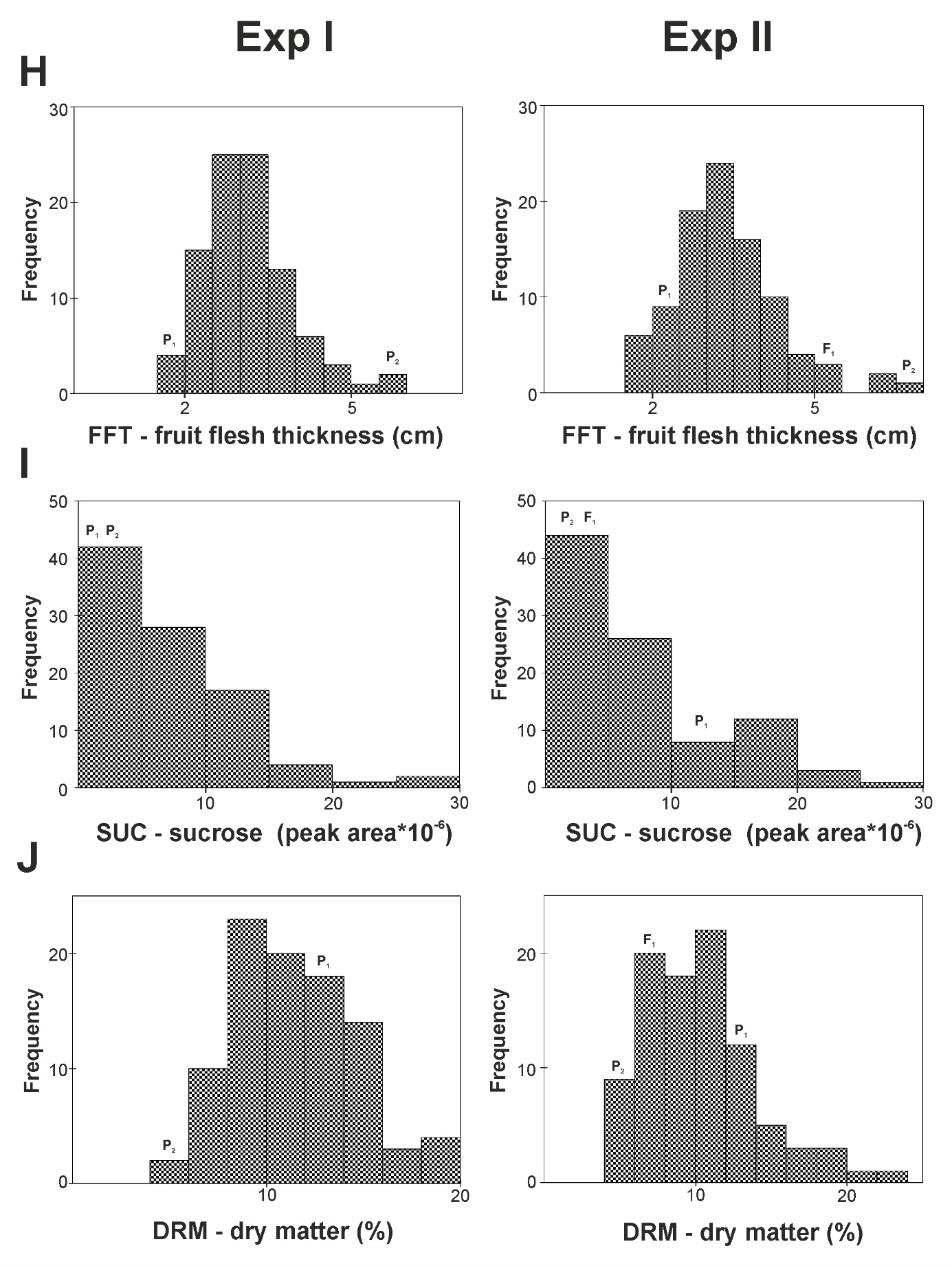


# Supplementary Figure S2.

HPLC profiles of measured sugars for parental lines and F_1_ in Exp 2. Peak represents: (1) fructose, (2) glucose, (3) sucrose. A - P_1_-802, B - P_2_-801 and C – F­_1_.


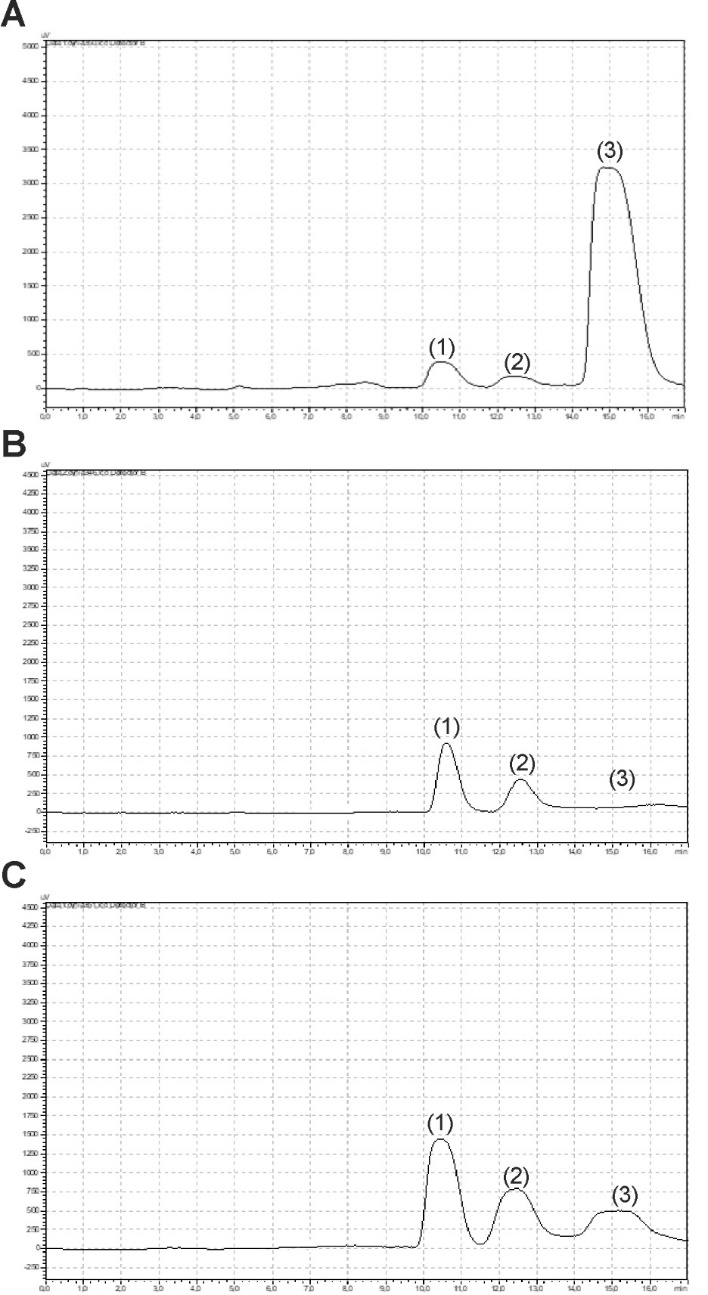

Supplement: Supplementary file 1 [file genes-11-00419-s001.zip › AppendixA_Kazminska_et_al_Suppl_Figures.docx]
